# High and growing disapproval of sex-selection technology in Australia

**DOI:** 10.1186/s12978-018-0577-5

**Published:** 2018-09-06

**Authors:** Rebecca Kippen, Edith Gray, Ann Evans

**Affiliations:** 1 School of Rural Health, Monash University, 26 Mercy St, Bendigo, VIC 3552 Australia; 2 School of Demography, The Australian National University, Building 9, Acton, ACT 2601 Australia

**Keywords:** Sex-selection technology, Australia, Gender, Sex ratio, IVF, Abortion

## Abstract

**Background:**

In Australia, the National Health and Medical Research Council has banned the use of assisted reproductive technology for social sex selection, but notes “there is limited research into the question of whether Australians support the use of sex selection for non-medical purposes”. This paper investigates Australian attitudes to sex-selection technology by different means (IVF, abortion, and a hypothetical pill), for different reasons (medical, family balancing, any reason), and by differing respondent characteristics (age, sex, education and religiosity).

**Methods:**

In 2007 and 2016, the Australian Survey of Social Attitudes (AuSSA) collected data on the attitudes of Australian adults to sex selection through IVF, abortion, and a hypothetical pill. We calculate population-weighted distributions and 95% confidence intervals of responses, and carry out logistic regressions to investigate the demographic characteristics of Australians who strongly disapprove of IVF or abortion for sex selection.

**Results:**

In 2016, around three-quarters of AuSSA respondents were opposed to legalising sex selection through IVF for any reason, or for family balancing for a second or third child. Thirty-seven per cent were opposed to IVF for medical sex selection. Two-thirds of respondents in both 2007 and 2016 disapproved or strongly disapproved of IVF for sex selection, while the proportion who strongly disapproved increased from 31 to 40%. Disapproval/strong disapproval of abortion for sex selection increased from 74 to 81% from 2007 to 2016, while strong disapproval alone rose from 44 to 55%. More than 70% of respondents in both 2007 and 2016 stated that a hypothetical pill for sex selection should not be legal. Our analysis finds that female, young, more-educated, and more religious respondents are more likely to strongly disapprove of sex selection via IVF or abortion, and that the increase in those who strongly disapprove from 2007 to 2016 is statistically significant.

**Conclusions:**

Australians generally disapprove of the use of sex-selection technology. If legislation is to be guided by community attitudes, then the prohibition against sex selection for non-medical purposes through assisted reproductive technology should be maintained.

## Plain English summary

In Australia, the use of reproductive technology to choose the sex of children is outlawed for other than medical reasons. Regarding this prohibition, the Australian National Health and Medical Research Council (NHMRC) noted in 2017 that “society’s readiness to accept a practice is a relevant and important consideration” and “there is limited research into the question of whether Australians support the use of sex selection for non-medical purposes”.

We address this question through analysis of a large nationally representative survey: the 2007 and 2016 Australian Survey of Social Attitudes. We find surprisingly low levels of support for the use of sex-selection technology, with most Australians disapproving of sex selection through IVF, abortion, and a hypothetical pill. This disapproval has strengthened over the period 2007 to 2016, with female, young, more-educated and more religious respondents more likely to strongly disapprove of sex selection via IVF or abortion.

We conclude that if legislation is to be guided by “society’s readiness to accept a practice”, as noted by the NHMRC, then the prohibition against sex selection for non-medical purposes through assisted reproductive technology should be maintained.

## Background

In Australia, the use of assisted reproductive technology (ART) to select the sex of children for non-medical reasons has been banned since 2004. In that year, the Australian National Health and Medical Research Council (NHMRC) introduced *Ethical Guidelines on the Use of Assisted Reproductive Technology in Clinical Practice and Research* [1]. These guidelines, under the section heading “Do not select sex for nonmedical purposes” stated:

Sex selection is an ethically controversial issue. The Australian Health Ethics Committee believes that admission to life should not be conditional upon a child being a particular sex. Therefore, pending further community discussion, sex selection (by whatever means) must not be undertaken except to reduce the risk of transmission of a serious genetic condition.

An Appendix to the 2004 guidelines listed three reasons for and three reasons against the use of sex selection for non-medical reasons, in order to “foster and assist community debate”. Reasons for were: family balancing, fulfilment of religious obligations or cultural expectations that require offspring of a particular sex, and the right to individual reproductive autonomy. Reasons against were: incompatibility with unconditional acceptance by parents of their children, possible expression of gender bias against girls, and possible sex ratio distortions leading to a “shortage of women for men to marry” [1].

In practice the sex selection prohibition applied to in vitro fertilisation (IVF) only, with preimplantation genetic diagnosis (PGD) used to determine embryo sex before implantation. At the time the prohibition was introduced in Australia in 2004, the practice of social sex selection via IVF was minimal. Three of the eight Australian states and territories had already legislated against social sex selection using IVF, and only a small number of ART clinics in the other states offered social sex selection [2]. In the year before the NHMRC guidelines came into force, only 90 babies across Australia and New Zealand were born as a result of ‘ART involving PGD’ (in the context of more than 300,000 total births), with PGD mostly used to test for genetic disorders, rather than for social sex selection [3, 4].

The NHMRC guidelines were revised in 2007 [5], and again in 2017 [6]. The prohibition against sex selection for non-medical reasons remained in place. In their 2017 revised guidelines, the NHMRC noted:

Many of the issues surrounding ART are as much social and political as they are ethical. With any controversial practice, society’s readiness to accept a practice is a relevant and important consideration. At the time of publication, there is limited research into the question of whether Australians support the use of sex selection for non-medical purposes.

This paper adds to debate and knowledge in this area by using a large national survey to assess support by Australians for the use of IVF to choose the sex of a child for medical reasons, for family balancing, or for any reason, since support may vary by the motive for sex selection. The NHMRC and some researchers have previously suggested that social sex selection for family balancing—where parents with children all of the same sex choose to have a child of the opposite sex—may be more socially and ethically acceptable than other motivations for social sex selection, such as choosing to have a first-born son or only sons [7–10].

We also investigate whether support for sex selection in Australia has changed over time, whether it varies by other methods of sex selection (sex-selective abortion or a hypothetical blue pill/pink pill) in contrast to IVF, or by demographic characteristics of the survey respondents. This updates a previous study conducted in 2007 [7].

## Data and methods

Data for this study are derived from the Australian Survey of Social Attitudes (AuSSA). AuSSA is an annual national postal survey of adult Australians chosen at random from the Australian Electoral Roll (enrolment on the Roll is compulsory for all Australian citizens aged 18 years and over). The survey is “Australia’s main source of data for the scientific study of the social attitudes, beliefs and opinions of Australians, how they change over time, and how they compare with other societies” [11]. In 2007 and 2016, AuSSA included the following questions on sex selection:

1. Do you approve or disapprove of the use of IVF technology to avoid characteristics of children such as: a certain sex?
  Strongly approve/Approve/Neither approve nor disapprove/Disapprove/Strongly disapprove/Don’t know
2. Do you approve or disapprove of the use of abortion to avoid having children with characteristics such as: a certain sex?
  Strongly approve/Approve/Neither approve nor disapprove/ Disapprove/Strongly disapprove/Don’t know
3. Suppose there was a medication available that enabled parents to choose the sex of their children. Couples simply had to take a blue pill to ensure the birth of a boy or a pink pill to ensure the birth of a girl. Do you think such a medication should be legally available?
  Yes/No/Don’t know
4. If you were planning to have children, would you take advantage of such a medication?
  Yes/No/Don’t know

Questions 3 and 4 were adapted from a question asked by Dahl et al. in a series of studies [12–14] in the early 2000s: “Suppose there was a medication enabling parents to choose the sex of their children. Couples simply had to ingest a blue pill to ensure the birth of a boy or a pink pill to ensure the birth of a girl. Would you take advantage of such a medication?”

Additional questions around whether IVF for sex selection should be legal, and under what circumstances, were asked in 2016 only:

5. Should IVF be legal in Australia for choosing a child’s sex for: medical reasons?
  Yes/No/Don’t know
6. Should IVF be legal in Australia for choosing a child’s sex for: family balancing - sex of second child different from first?
  Yes/No/Don’t know
7. Should IVF be legal in Australia for choosing a child’s sex for: family balancing - sex of third child different from first two?
  Yes/No/Don’t know
8. Should IVF be legal in Australia for choosing a child’s sex for: any reason?
  Yes/No/Don’t know

In 2007, three versions of AuSSA—A, B and C—with different sets of questions were sent out. Version A contained the above questions 1 to 4 on sex selection, and was sent to 6666 people on the Australian Electoral Roll. Responses totalled 2781, or 42% [15]. In 2016, one version of the survey was sent to 5000 people, with a response rate of 25% (1267). Responses are weighted to national counts of the adult citizen population by age, sex, and education.

**Fig. 1:**
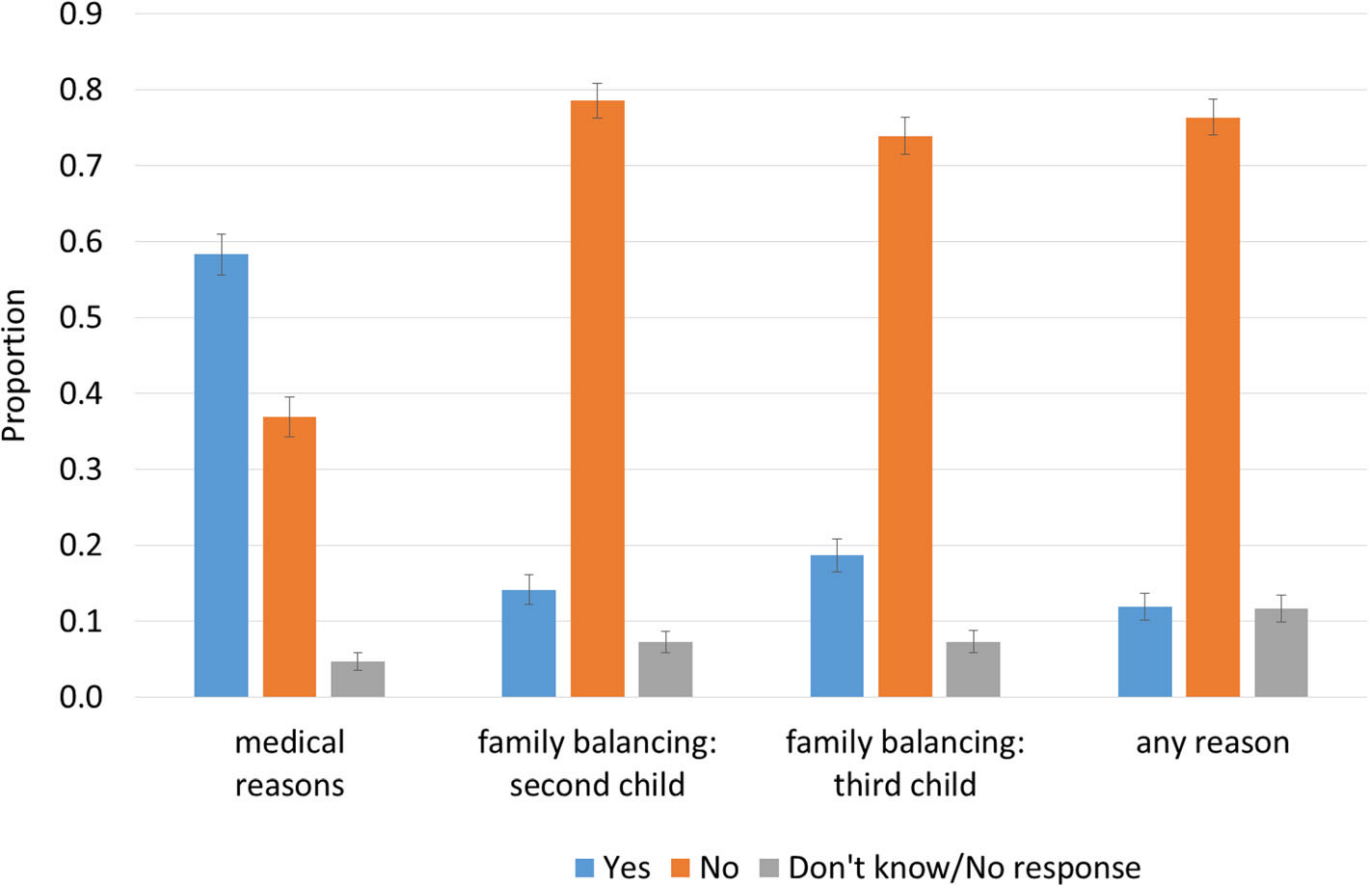
Responses to “Should IVF be legal in Australia for choosing a child’s sex for: medical reasons; family balancing—sex of second child different from first; family balancing—sex of third child different from first two; any reason”. Note: Weighted to population characteristics. 95% confidence intervals shown. Source: Australian Survey of Social Attitudes, 2016, *n* = 1267

**Fig. 2:**
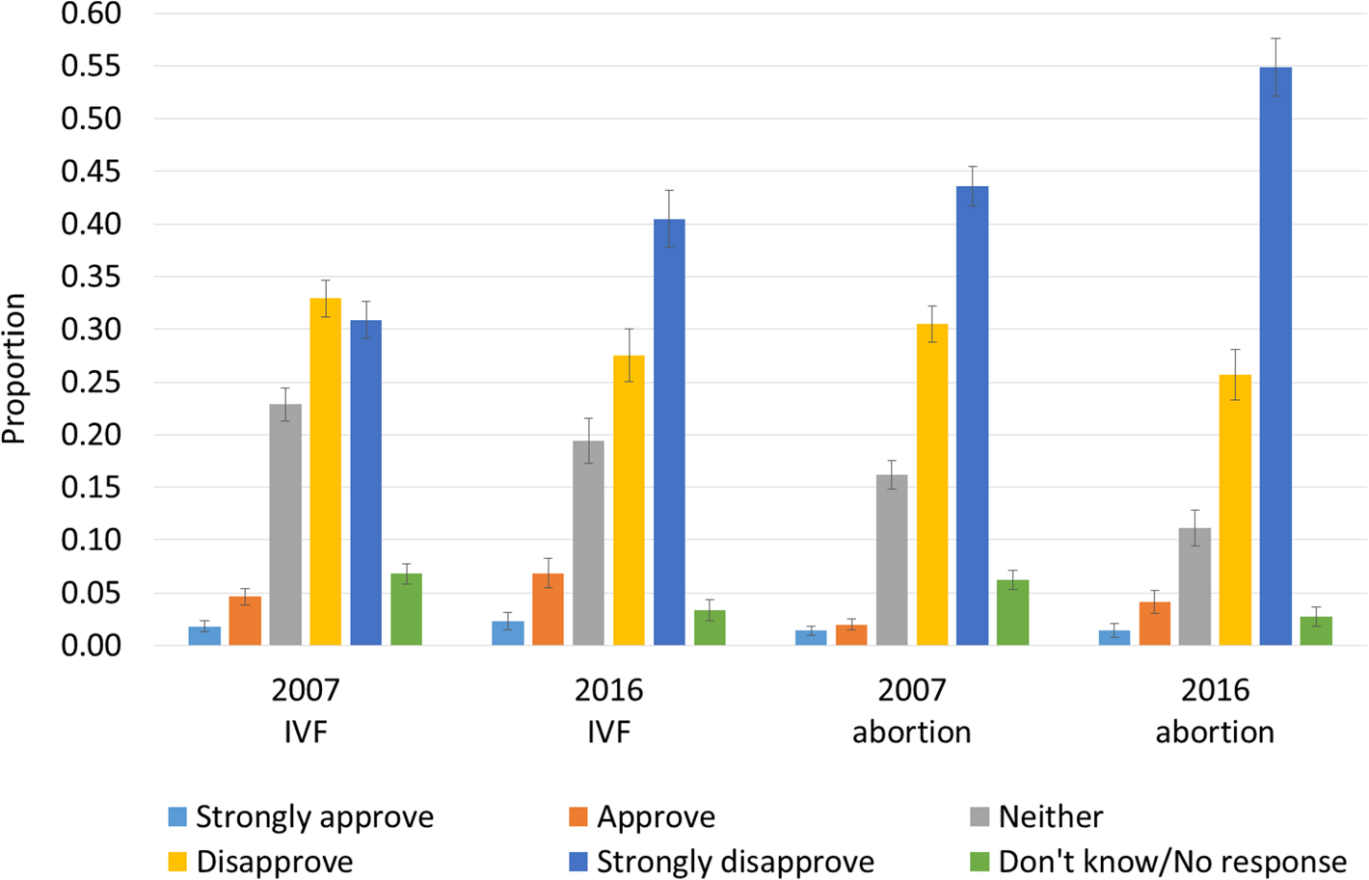
Responses to “Do you approve or disapprove of the use of IVF technology to avoid characteristics of children such as: a certain sex” and “Do you approve or disapprove of the use of abortion to avoid characteristics of children such as: a certain sex”. Note: Weighted to population characteristics. 95% confidence intervals shown. Source: Australian Survey of Social Attitudes, 2007 and 2016, *n* = 4048

**Fig. 3:**
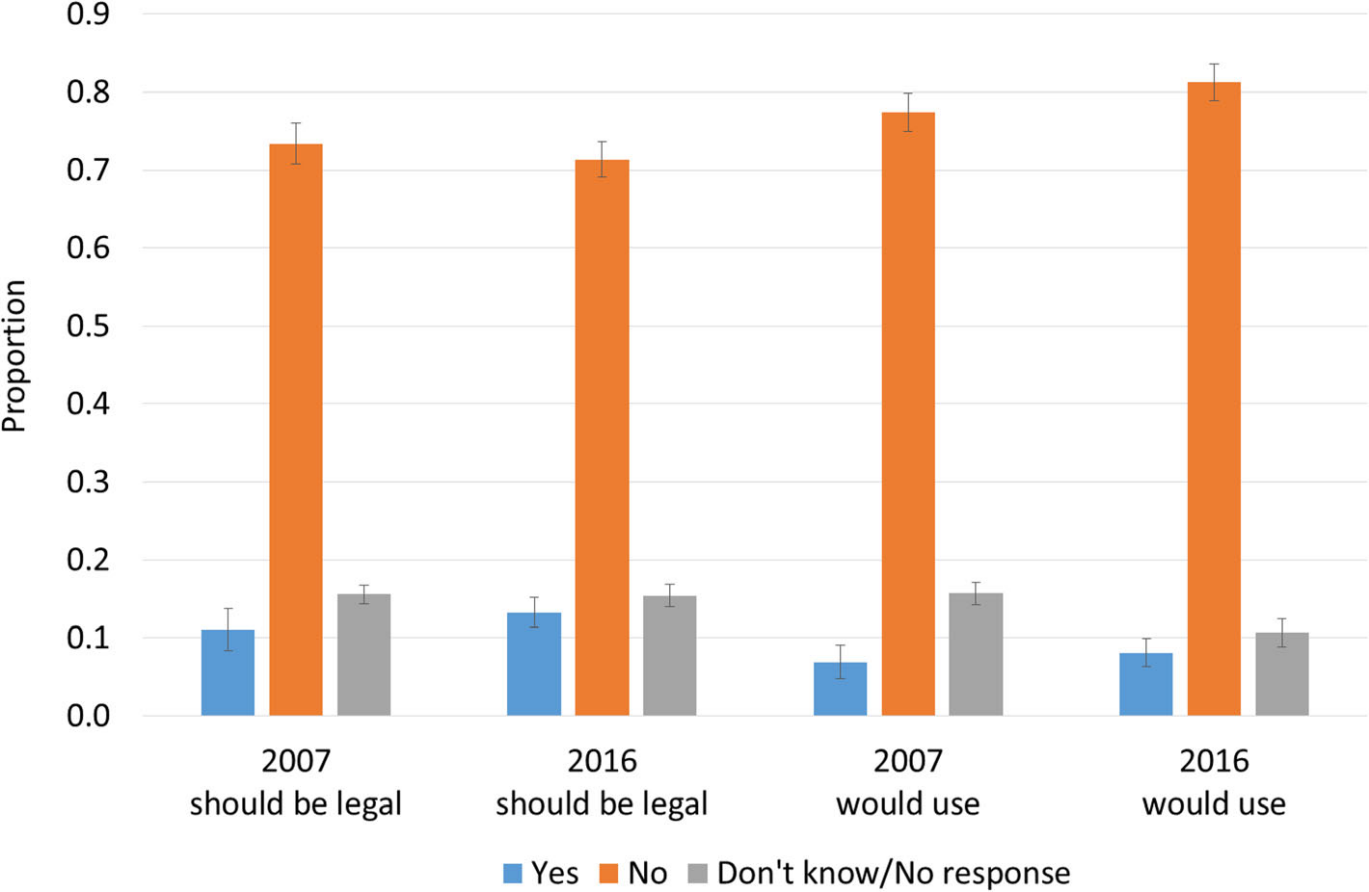
Responses to “Suppose there was a medication available that enabled parents to choose the sex of their children…Do you think such a medication should be legally available?” and “If you were planning to have children, would you take advantage of such a medication?”. Note: Weighted to population characteristics. 95% confidence intervals shown. Source: Australian Survey of Social Attitudes, 2007 and 2016, n = 4048

We calculated population-weighted distributions of sample survey responses to each of the eight questions, shown in Figs. 1, 2 and 3. These figures also show 95% confidence intervals for the population proportion for each response category, shown as error bars for each proportion, and calculated with z-scores on the assumption the data were normally distributed. Non-overlapping confidence intervals indicate that differences between proportions are statistically significant.

We also constructed two logistic regression models, testing whether “Strong disapproval” of IVF for sex selection, or “Strong disapproval” of abortion for sex selection, varies by age, sex, education or religious attendance, and whether the level of “Strong disapproval” changed in the Australian population between 2007 and 2016 (Table 1). These models show odds ratios, 95% confidence intervals, *p*-values, predicted probabilities, and sample n for each category.

**Table 1:** Logistic regressions. Responses to “Do you approve or disapprove of the use of IVF technology [abortion] to avoid characteristics of children such as: a certain sex”. Strongly disapprove (vs Disapprove, Neither, Approve, Strongly Approve), by survey year, and respondent sex, age, education and religious attendance

Sample characteristics	IVF					Abortion				
	Odds ratio	95% CI	*p*-value	Pred. prob.	n	Odds ratio	95% CI	*p*-value	Pred. prob.	n
SEX										
Ref: Male	1.00			0.378	1836	1.00			0.506	1845
Female	1.39	(1.21–1.59)	0.000	0.457	1945	1.31	(1.14–1.49)	0.000	0.572	1959
AGE										
Ref: 17–34 years	1.00			0.470	666	1.00			0.686	672
35–49 years	0.95	(0.77–1.16)	0.606	0.457	989	0.77	(0.63–0.94)	0.012	0.627	987
50–64 years	0.95	(0.78–1.16)	0.611	0.457	1215	0.61	(0.50–0.75)	0.000	0.572	1223
65+ years	0.57	(0.46–0.72)	0.000	0.337	878	0.39	(0.31–0.49)	0.000	0.462	886
EDUCATION										
Ref: Bachelor degree or above	1.00			0.536	1030	1.00			0.649	1038
Other post-school qualification	0.73	(0.62–0.86)	0.000	0.457	1588	0.72	(0.62–0.85)	0.000	0.572	1590
Year 12 or equivalent	0.71	(0.55–0.91)	0.007	0.450	377	0.64	(0.50–0.82)	0.000	0.543	380
Year 10/11 or equivalent	0.59	(0.46–0.74)	0.000	0.403	500	0.50	(0.40–0.63)	0.000	0.479	505
Below Year 10	0.56	(0.41–0.77)	0.000	0.393	261	0.41	(0.31–0.56)	0.000	0.433	264
RELIGIOUS ATTENDANCE										
Ref: Never	1.00			0.483	1465	1.00			0.601	1471
Once a year or less frequently	0.90	(0.76–1.07)	0.234	0.457	1074	0.89	(0.75–1.05)	0.157	0.572	1074
Several times a year	0.90	(0.72–1.11)	0.318	0.455	497	0.97	(0.79–1.20)	0.806	0.595	499
Once a month or more	1.29	(1.06–1.57)	0.010	0.547	651	1.27	(1.05–1.54)	0.016	0.656	664
YEAR										
Ref: 2007	1.00			0.365	2596	1.00			0.472	2613
2016	1.47	(1.26–1.70)	0.000	0.457	1216	1.50	(1.29–1.73)	0.000	0.572	1221
Total sample					3812					3834

Source: Australian Survey of Social Attitudes, 2007 and 2016

Ref: Reference category

95% CI: 95% confidence interval for odds ratios

Pred. prob.: Predicted probability. Other characteristics set at Female, 50–64 years, Other post-school qualification, Once or year or less frequently, 2016

Don’t know/No response cases for the dependent variable are excluded from analysis

Don’t know/Other/No response cases for independent variables are included in the analysis but not shown

*p*-values of less than 0.05 are considered significant

## Results

### Sex selection through IVF for medical reasons, family balancing or any reason

The 2016 Australian Survey of Social Attitudes asked, for the first time, whether IVF should be legal in Australia for choosing a child’s sex, and under what circumstances. Results are shown in Fig. 1.

Fifty-eight per cent of Australian adults believe that sex selection through IVF should be legal for medical reasons. Thirty-seven per cent of respondents answered in the negative, while 5% did not know, or did not give a response. In contrast, around three-quarters of Australians are opposed to legalising sex selection through IVF for any reason, for choosing the sex of a second child different from the first, or for choosing the sex of a third child different from the first two.

Only 14% believe IVF should be legally available for choosing the sex of a second child different from the first, and 19% for family balancing for a third child. Twelve per cent support the legalisation of sex selection through IVF for any reason, with an equal proportion declining to answer this question or giving a “Don’t know” response. For the two family balancing options, 7% of survey participants gave a ‘Don’t know’ response, or did not provide a response (Fig. 1). The differences between the yes/no responses for legalisation of sex selection for medical reasons, and the yes/no responses for legalisation of sex selection for family balancing or for any reason, are statistically significant; Australians are much more likely to be favour of sex selection through IVF for medical reasons, and much more likely to be opposed to sex selection through IVF for other than medical reasons.

### Disapproval of IVF and abortion for choosing a child’s sex

Questions around approval or disapproval of IVF or abortion for choosing the sex of a child were asked in AuSSA in 2007 and 2016. Population-weighted responses are shown in Fig. 2. For all four groupings, the largest response categories are “Disapprove” and “Strongly disapprove”, with “Strongly disapprove” the largest category for IVF in 2016, and abortion in both 2007 and 2016.

Around two-thirds of respondents in both 2007 and 2016 disapproved or strongly disapproved of the use of IVF for sex selection. Those who disapproved or strongly disapproved of the use of abortion for sex selection increased from 74% in 2007 to 81% in 2016.

The combined Approve/Strongly approve category increased for IVF between 2007 and 2016, from 6% to 9%, and for abortion from 3% to 6%. However, these increases were not statistically significant. The proportion who selected “Neither approve nor disapprove” or “Don’t know”, or who gave no response, fell for both IVF and abortion between 2007 and 2016.

The most significant shift in attitudes to the use of IVF or abortion for sex selection was in the strengthening of the “Strongly disapprove” response between 2007 and 2016. Those who strongly disapprove of the use of IVF for sex selection increased from 31% to 40%, and for abortion, from 44% to 55%, between 2007 and 2016 (Fig. 2). This result is statistically significant, as indicated by the non-overlapping confidence intervals.

### Demographic characteristics associated with strong disapproval of the use of sex selection through IVF or abortion

We constructed two logistic regression models to determine whether particular groups of Australians were more likely to strongly disapprove of the use of sex-selective IVF or abortion. Characteristics considered were sex, age, education, and religious attendance (as a proxy for religiosity). In these models, we also included a marker for survey year to assess whether the increase in strong disapproval from 2007 to 2016—seen in Fig. 2—remained after controlling for respondent characteristics.

Results of the two models are shown in Table 1. Odds ratios greater than 1.00 indicate that the population group in question has a higher proportion who strongly disapprove compared to the reference category, while odds ratios of less than 1.00 indicate a lower level of strong disapproval in comparison to the reference category. *P*-values of less than 0.05 are considered significant. The predicted probabilities of a person with the specified characteristics “strongly disapproving” of sex-selective IVF or abortion are also shown.

In the first model, those more likely to strongly disapprove of IVF for sex selection are women (predicted probability = 46%, *p* = 0.000, compare men), young people aged 17–34 years (predicted probability = 47%, *p* = 0.000, compare 65+ years), those with a bachelor degree or above (predicted probability = 54%, *p* = 0.000, compare other education levels), and those who attend religious services once a month or more (predicted probability = 55%, *p* = 0.010, compare never). Levels of strong disapproval are higher for sex-selective abortion than for sex-selective IVF (Model 2) but patterns are similar to those found for IVF. Again, those more likely to strongly disapprove of abortion for sex selection are women (predicted probability = 57%, *p* = 0.000, compare men), young people aged 17–34 years (predicted probability = 69%, *p* = 0.000, compare other age groups), those with a bachelor degree or above (predicted probability = 65%, *p* = 0.000, compare other education levels), those who attend religious services once a month or more (predicted probability = 66%, *p* = 0.016, compare never). Those who strongly disapprove of IVF for sex selection are essentially a subset of those who strongly disapprove of abortion for sex selection (not shown). As in Fig. 2, the proportion of the population who strongly disapprove of IVF or abortion for sex selection increased significantly between 2007 and 2016 (Table 1; *p* = 0.000).

### Attitudes to a hypothetical blue pill/pink pill for choosing a child’s sex

In the early 2000s, Dahl et al. ran a series of surveys on sex selection in Germany, the United Kingdom, and the United States [12–14]. One of the questions asked was whether the respondent would take advantage of a hypothetical blue pill or pink pill, were such technology available, to ensure the birth of a son or daughter. We employed this question in the Australian Survey of Social Attitudes, and also extended it to ask whether respondents believed this medication should be legal, were it to exist. The rationale for these questions is to separate out those who may be opposed to the *method* of sex selection (such as IVF or abortion), from those opposed to the *motivation* (e.g. gender discrimination) or *outcome* (e.g. sex-ratio imbalance) of sex selection. Weighted responses to these two questions, for 2007 and 2016, are shown in Fig. 3.

As with IVF and abortion for sex selection, most Australians are opposed to a hypothetical blue pill/pink pill option. More than 70% believe that such technology should not be legally available, around 15% did not know or gave no response, and only 12% responded that the medication should be legal. If such a medication *were* available, 8% of respondents stated that they would take advantage of it, and around 80% stated that they would not. Response distributions were similar between 2007 and 2016.

## Discussion

This analysis of data from the 2007 and 2016 Australian Survey of Social Attitudes shows high and strengthening disapproval of sex selection via IVF or abortion. Most Australians believe that social sex selection via IVF should not be legal, even if it is for family balancing for a second or third child. However most are in favour of sex selection for medical reasons. In addition, most Australians believe that a hypothetical blue/pink pill for sex selection should not be legal.

From 2007 to 2016, there has been a shift from ‘Neither approve nor disapprove’ and ‘Disapprove’ to ‘Strongly disapprove’ for the use of IVF or abortion to choose the sex of a child. This shift is statistically significant. That is, disapproval of sex selection via IVF or abortion has strengthened in the Australian population over the past decade. This change is not indicative of strengthening opposition to IVF and abortion in general. Other research that we have conducted based on 2007 and 2016 AuSSA data (not shown) indicates low levels and little change in disapproval for IVF and abortion for reasons other than social sex selection, such as to avoid a physical disability. Rather, results of this study are reflective of strengthening opposition to choosing to have a child on the basis of whether that child is male or female.

Those most likely to strongly disapprove of IVF or abortion for sex selection are women, young adults, university educated, and more religious respondents. These would seem to be strange bedfellows. An area for further research is to determine why these groups are more opposed than are others to sex selection. It may be that women, young adults and the university educated see sex selection as problematic because of the perceived implication that the process equates sex with gender, with parents selecting the sex of their offspring because of normative stereotypes of gender. If this is the case, then opposition is likely to continue to strengthen in the future. Conversely, more religious respondents may oppose sex selection because of the methods employed. Other studies in Australia and internationally find that the more religious are more likely to oppose the use of IVF or abortion for any reason, not just for sex selection [16–18].

The findings of high opposition to social sex selection—whatever the means—are in line with international results. A 2006 review of research internationally on “Attitudes towards sex selection for non-medical reasons” [19] identified 21 relevant studies conducted 1971–2005, 16 on US populations, 4 from Germany and 1 from the United Kingdom. All these studies found majority disapproval for the use of sex selection technology, both when questions were framed for personal use, and whether such technology should be generally available or made legal. In accordance with the findings of the current study, the review found stronger disapproval for personal use compared to general availability, and variation by proposed method, such that disapproval was higher for sex-selective abortion than for sex-selective IVF and for a hypothetical blue/pink pill. More recent studies have similar findings [12–14, 20–22], including one for Australia [23].

Our research adds to this international literature through investigating, for Australia, attitudes to sex selection for different reasons—medical, family balancing, any reason—changes in attitudes over time, and the demographic characteristics of those more likely to be opposed to sex selection.

What are the implications for Australian law of opposition to social sex selection? Currently, the blue/pink pill scenario for sex selection is still a hypothesis. It may be decades, if ever, before a sex-selective pill becomes a reality. And, in practice, early-term abortion is available on request in Australia, although what constitutes ‘early-term’ varies geographically as abortion law is state-based. Early maternal blood tests are now available that can determine fetal sex [24], and there is some evidence that sex-selective abortion is occurring in Australia, albeit on a very limited basis [25].

Any legal implications are most relevant for the use of IVF for sex selection. As noted in the Introduction, Australia’s *Ethical Guidelines on the Use of Assisted Reproductive Technology in Clinical Practice and Research*, which have the force of law, prohibit the use of social sex selection “by whatever means”. In practice, this prohibition had the effect of shutting down the use of IVF for social sex selection in Australian fertility clinics.

One school of thought is that legislation should be guided by community attitudes. This seems to be the position of the NHMRC, which in its 2017 revision of the *Ethical Guidelines* stated that “With any controversial practice, society’s readiness to accept a practice is a relevant and important consideration.” Our study finds that Australians are opposed to the use of sex selection, including via IVF, except where its use is recommended for medical reasons. Although the NHMRC has previously suggested that social sex selection for family balancing may be more acceptable than for other reasons [8, 9], our research shows that opposition by Australians to social sex selection is fairly consistent whether it is for family balancing for the second child, or third child, or for any reason.

A second school of thought is the legality of social sex selection should be guided by its potential for harm. However there is no consensus among ethicists and others about whether allowing social sex selection would result in harm or not. Possible harms that have been discussed include: distortions in the sex ratio of births, particularly if sons are preferred, as currently happens in some countries [1, 26, 27]; distortions in the sex ratio of first births, if boys and girls are equally desired, but there is a preference for first-born sons [7, 28, 29]; sex selection as expressed discrimination against females [1, 26, 27]; sex selection as a manifestation of normative gender stereotypes both within individual families and within society at large [26, 30, 31]; and sex selection as a “slippery slope” to designer babies [26, 27, 32].

The arguments for and against harm are equally vehement, and we are not in a position to judge most of them here. However, as demographers, we can comment on the first two possible harms mentioned: distortions in the overall sex ratio at birth, and in the sex ratio of first births. Previous research on parental behaviour and attitudes indicates that a change in the sex ratio of all births is unlikely in the Australian context, as sons and daughters are equally desired, with parents overall wishing for a mix of boys and girls [29, 33–35].

However, a previous study has found that a significant minority of Australians (40%) intending to have a first child in the future express a preference for the sex of that child, with first-born sons chosen over first-born daughters by more than two to one [34]. If social sex selection was permitted for any reason in Australia, it may lead to a distortion in the sex ratio of first-born children, with many more first-born boys than first-born girls. This is of concern, since research has found that first-borns are more likely to have attributes such as higher levels of intelligence, education and general achievements [29].

## Conclusions

Assisted reproductive technology for social sex selection is banned in Australia. The prohibiting body noted in 2017 that community acceptance of the practice of social sex selection would be an important consideration in future regulatory change, but, currently, “there is limited research into the question of whether Australians support the use of sex selection for non-medical purposes” [6].

This study addresses this question, finding from analysis of a recent large national representative survey that around three-quarters of Australians believe the use of IVF for non-medical sex selection should not be legal, whether it be for family balancing or for any reason. In contrast, most Australians (58%) believe that sex selection through IVF should be legal for medical reasons.

Opposition to sex selection via IVF has strengthened over the past decade (as has opposition to sex selection via abortion). Clearly, the Australian community does not accept the practice of social sex selection. If regulatory change is to be guided by societal attitudes, then the prohibition against sex selection for non-medical reasons should remain in place.

If, instead, potential harms are paramount, then social sex selection should remain prohibited if there is a demonstrable harm from its use. Many potential harms have been raised, with a focus in this paper’s discussion on possible distortions in the sex ratio at birth, if widespread social sex selection is used to choose many more sons than daughters, as happens in some other countries [1, 7]. Is such concern valid? Previous research finds that Australian parents have an overall equal preference for sons and daughters [29, 33–35], but that a large minority of intending parents have a preference for a first-born boy [34].

Thus, the legalisation of social sex selection is unlikely to lead to a surfeit of sons in Australia, but could result in a preponderance of first-born boys. Only allowing sex selection for family balancing after the first child would obviate any possible sex ratio distortions for first-borns. However such a recommendation depends on how much weight should be given to community attitudes, which in Australia are clearly against the use of social sex selection—even for the second or third child—with disapproval strengthening over the past decade.

## Strengths and limitations of the study

A major strength of this study is that it is based on analysis of a large, nationally representative random survey of adult Australian citizens—the Australian Survey of Social Attitudes (AuSSA). The sample size was 2781 in 2007 and 1267 in 2016. Response rates fell over this period from 42% in 2007 to 25% in 2016. The collection of demographic characteristics of respondents means the data can be weighted to national population counts by age, sex and education, decreasing non-response bias. However, non-response bias due to other characteristics such as ethnicity or religion may remain. Because AuSSA is a broad survey of social attitudes, non-response is unlikely to be related to a reluctance to answer questions around the study topic [36]. Another limitation of this study is that we are unable to directly ascertain the reasoning behind the stated attitudes.
